# Group 2 innate lymphoid cells are elevated and activated in chronic rhinosinusitis with nasal polyps

**DOI:** 10.1002/iid3.161

**Published:** 2017-04-19

**Authors:** Julie A. Poposki, Aiko I. Klingler, Bruce K. Tan, Pejman Soroosh, Homayon Banie, Gavin Lewis, Kathryn E. Hulse, Whitney W. Stevens, Anju T. Peters, Leslie C. Grammer, Robert P. Schleimer, Kevin C. Welch, Stephanie S. Smith, David B. Conley, Joseph R. Raviv, James G. Karras, Omid Akbari, Robert C. Kern, Atsushi Kato

**Affiliations:** ^1^ Division of Allergy‐Immunology Department of Medicine Northwestern University Feinberg School of Medicine Chicago Illinois USA; ^2^ Department of Otolaryngology Northwestern University Feinberg School of Medicine Chicago Illinois USA; ^3^ Janssen Research and Development San Diego California USA; ^4^ Division of Otolaryngology‐Head and Neck Surgery, NorthShore University HealthSystem, The University of Chicago Pritzker School of Medicine Evanston Illinois USA; ^5^ Department of Molecular Microbiology and Immunology, Keck School of Medicine University of Southern California Los Angeles California USA

**Keywords:** Chronic rhinosinusitis, ILC2, innate lymphoid cells, nasal polyp, Type 2 inflammation

## Abstract

**Background:**

Chronic rhinosinusitis (CRS) with nasal polyps (CRSwNP) is characterized by type 2 inflammation with high levels of Th2 cytokines. Although T helper cytokines are released from T cells, innate lymphoid cells (ILC) are also known to produce high levels of the same cytokines. However, the presence of various types of ILC in CRS is poorly understood.

**Objective:**

The objective of this study was to fully characterize the presence of all ILC subsets in CRS and to identify phenotypical differences of group 2 ILC (ILC2) in CRSwNP compared to ILC2 from non‐type 2 inflamed areas.

**Methods:**

We investigated the presence of ILC subsets in peripheral blood mononuclear cells (PBMC) from healthy subjects, tonsil tissue, ethmoid tissue from control subjects and patients with non‐polypoid CRS (CRSsNP) and CRSwNP, as well as nasal polyp (NP) tissue from CRSwNP by flow cytometry. Sorted ILC2 were cultured in the presence and absence of IL‐33 and production of IL‐5 and IL‐13 was assessed by Luminex.

**Results:**

We found that all ILC subsets were present in NP but ILC2 were dominant and significantly elevated compared to PBMC, tonsil, CRSsNP, and normal sinus tissue. We also found that inducible T‐cell co‐stimulator (ICOS) and side scatter were increased and CD127 was down‐regulated in ILC2 from NP compared to blood or tonsil ILC2. Thymic stromal lymphopoietin, IL‐7, and IL‐33 were able to down‐regulate expression of CD127 and increase side scatter in blood ILC2. Furthermore, sorted NP ILC2 but not blood ILC2 spontaneously released type 2 cytokines including IL‐5 and IL‐13.

**Conclusions and Clinical Relevance:**

These results suggest that ILC2 are not only elevated but also activated in CRSwNP in vivo and that ILC2 may play important roles in the type 2 inflammation in CRSwNP.

## Introduction

Chronic rhinosinusitis (CRS) is characterized by the presence of high levels of T helper (Th) cytokines and polypoid CRS (CRSwNP) is characterized by type 2 inflammation with high levels of Th2 cytokines including IL‐5 and IL‐13 1, 2, 3. Recent studies indicate that T helper cytokines are not only produced from T cells but also from other immune cells including innate lymphoid cells (ILC), a minor population of immune cells 4. Therefore, Th2 cytokines are now also described as type 2 cytokines. Although morphologically ILC are similar to lymphocytes, they lack antigen receptors and lineage (Lin) markers yet produce high levels of T helper cytokines. Like T helper cells, ILC can be classified into three subsets; group 1 ILC (ILC1), ILC2, and ILC3 4. ILC1 are characterized by the expression of T‐bet and production of the type 1 cytokine IFN‐γ. ILC2 express GATA3 and produce type 2 cytokines including IL‐5 and IL‐13. ILC3 are characterized by the expression of RORγt and the production of type 3 (also known as type 17) cytokines including IL‐17A and IL‐22. ILC3 can be further divided into two subsets based on the expression of natural cytotoxicity receptors (NCR) including NKp44. Recent studies indicate that ILC2 are significantly elevated in nasal polyps (NPs) from patients with CRSwNP 5, 6, 7, 8, 9. However, although the T cell population in CRS is well investigated 10, 11, the presence of ILC subsets, especially ILC1 and ILC3, in CRS is poorly understood. Furthermore, the phenotypic differences between ILC2 from type 2 inflamed tissues (NP in CRSwNP) and non‐type 2 areas (tonsil and blood from healthy subjects) are largely unknown. In the present study, we have fully characterized the presence of all ILC subsets in CRS and have identified phenotypical differences between ILC2 in NP compared to ILC2 from non‐type 2 inflamed areas.

## Materials and Methods

### Patients and tissue collection

Patients with CRS were recruited from the Otolaryngology clinic and the Northwestern Sinus Center of Northwestern Medicine. All patients met the criteria for CRS as defined by the European Position Paper on Rhinosinusitis and Nasal Polyps 2012 1. Patients with an established immunodeficiency, pregnancy, coagulation disorder or diagnosis of Churg‐Strauss syndrome or cystic fibrosis were excluded from the study. During endoscopic sinus surgery, ethmoid tissue was collected from CRS without NPs (CRSsNP) and CRSwNP, and NP tissue was collected in patients diagnosed with CRSwNP. Disease‐free ethmoid sinus tissues from normal control patients without a history of CRS were obtained during transnasal endoscopy skull base procedures. Tonsil tissue was obtained when patients underwent tonsillectomy for chronic tonsillitis, recurrent acute tonsillitis, or obstructive sleep apnea as collected by faculty of the Otolaryngology Department of Northwestern Medicine. Patients were skin‐tested for pollens, dust mites, pets, molds, and cockroach using Hollister‐Stier (Spokane, WA) extracts. Several patients were taking a variety of medications, including corticosteroids. Details of patients’ characteristics are included in Table 1. All subjects signed informed consent and the study was approved by the Institutional Review Board of Northwestern University Feinberg School of Medicine (IRB Project Number: STU00200416). Human buffy coats (American Red Cross, St. Paul, MN) and human peripheral blood leuko paks (STEMCELL Technologies, Vancouver, BC, Canada) were obtained from healthy subjects for isolation of peripheral blood mononuclear cells (PBMC).

**Table 1:** Subject characteristics

	Tonsil (*n* = 21) *n* (%)	Control (*n* = 5) *n* (%)	CRSsNP (*n* = 13) *n* (%)	CRSwNP (*n* = 9) *n* (%)	CRSwNP NP (*n* = 25) *n* (%)
Female	7 (33)	3 (60)	9 (69)	5 (56)	7 (28)
Atopy	8 (38)	1 (20)	8 (62)	6 (67)	19 (76)
Asthma	6 (29)	0 (0)	4 (31)	4 (44)	17 (68)
Aspirin sensitivity	0 (0)	0 (0)	0 (0)	1 (11)	6 (24)
Nasal steroid	3 (14)	0 (0)	5 (38)	3 (33)	9 (36)
Inhaled steroid	0 (0)	0 (0)	1 (8)	2 (22)	4 (16)
Oral steroid	0 (0)	1 (20)	1 (8)	2 (22)	9 (36)
Age (y), median (range)	33* (20–63)#	54 (19–72)	50 (18–72)	61 (33–69)	51 (24–80)

*Median, #(range).

### Cell isolation and flow cytometric analysis

Cells were isolated from tonsil and sinus tissue using a previously described method 12, 13. We obtained 759 ± 296 million live cells (tonsil, *n* = 21), 0.35 ± 0.21 million live cells (control, *n* = 5), 1.21 ± 0.69 million live cells (CRSsNP, *n* = 13), 3.96 ± 1.68 million live cells (CRSwNP ethmoid, *n* = 9) and 42.4 ± 10.4 million live cells (NP, *n* = 25) from 1847 ± 360 mg, 107 ± 48 mg, 108 ± 19 mg, 206 ± 64 mg, and 1630 ± 391 mg tissue, respectively.

Cells were first treated with Aqua LIVE/DEAD fixable dead cell staining reagent (Invitrogen, Carlsbad, CA, USA) as a live/dead discriminator. Cells were then incubated with an Fc Block reagent (Miltenyi Biotec, San Diego, CA, USA) for 10 min at 4°C in the dark. All antibodies were obtained from BioLegend (San Diego, CA, USA), unless otherwise stated. The following antibodies and dilutions were used to stain the surface of the cells: FITC anti‐human Lineage Cocktail (CD3, CD14, CD16, CD19, CD20, CD56, 1:20), 2 μg/ml FITC anti‐FcϵRIa (AER‐37), 4 μg/ml FITC anti‐CD11c (Bu15), 0.25 μg/ml Alexa Fluor 700 anti‐CD45 (HI30), 5 μg/ml Alexa Fluor 647 anti‐CRTH2 (Bim16), 2.25 μg/ml PE/Cy7 anti‐CD127 (A019D5), 5 μg/ml APC/Cy7 anti‐CD161 (HP‐3G10), PE/CF594 anti‐CD117 (yb5.b8, 1:20, BD Biosciences, San Jose, CA, USA), 2.5 μg/ml PE anti‐NKp44 (P44‐8), 5 μg/ml Brilliant Violet 421 anti‐KLRG1 (2F1/KLRG1), 2 μg/ml PerCP/Cy5.5 anti‐ICOS (C398.4A), 2.5 μg/ml PerCP/Cy5.5 anti‐CD4 (RPA‐T4), and 1.25 μg/ml PerCP/Cy5.5 anti‐CD5 (L17F12). Cells were stained for 30 min at 4°C in the dark, and washed with MACS buffer (Miltenyi Biotech). Cells were fixed with a BD Cytofix/Cytoperm Kit, resuspended in MACS buffer and stored at 4°C in the dark before analysis on an LSRII (BD Biosciences). For the detection of intracellular CD127, fixed cells were incubated with 0.6 μg/ml PE anti‐CD127 (ebioRDR5, eBioscience) in Perm/wash buffer (BD Biosciences) for 30 min at 4°C in the dark. A total of 4,983,000 ± 538,915 events (PBMC), 3,981,000 ± 322,800 events (tonsil), 146,800 ± 61,400 events (control), 151,900 ± 41,600 events (CRSsNP), 499,700 ± 199,100 events (CRSwNP ethmoid) or 1,418,000 ± 243,200 events (CRSwNP) were collected. A minimum of 3,000,000 (PBMC), 1,000,000 (tonsil), 19,000 (control), 15,000 (CRSsNP), 57,000 (CRSwNP ethmoid), or 30,000 (NP) events was collected for each sample. The frequency of CD45^+^ cells within the live cell population was 99.8 ± 0.05% (PBMC), 99.9 ± 0.02% (tonsil), 77.3 ± 6.81% (control), 85.2 ± 2.12% (CRSsNP), 91.8 ± 2.21% (CRSwNP ethmoid) and 95.2 ± 0.65% (NP) and it was significantly higher in CRSwNP (*p* < 0.05) and in NP (*p* < 0.001) than in CRSsNP. The frequency of granulocytes (SSC^high^, eosinophils plus neutrophils) in CD45^+^ cells was 0.488 ± 0.154% (PBMC), 0.454 ± 0.073% (tonsil), 23.9 ± 6.60% (control), 12.2 ± 2.97% (CRSsNP), 37.1 ± 5.59% (CRSwNP), and 59.0 ± 2.35% (NP). All analysis and compensation were performed with FlowJo software, version 10.1 (TreeStar, Ashland, OR), and each experiment contained the proper single‐stained control beads (BD Biosciences and eBioscience) and fluorescence minus one (FMO) negative controls.

### ILC2 sorting and real‐time RT‐PCR

Details can be found in the Materials and methods section in the Supporting information.

### Cell culture

PBMC were suspended in RPMI 1640 medium (Invitrogen) supplemented with 10% FBS (Invitrogen), 100 U/ml penicillin, and 100 µg/ml streptomycin (Invitrogen), and were cultured in the presence or absence of 10 ng/ml TSLP (R&D systems, Minneapolis, MN) or 10 ng/ml IL‐7 (R&D systems) for two days. The expression of CD127 on ILC2 was detected by a CytoFLEX (Beckman Coulter, Indianapolis, IN). Data analysis was performed with FlowJo software.

Sorted ILC2 (10,000 cells/ml) were suspended in RPMI 1640 medium supplemented with 25 IU/ml IL‐2 (Prometheus, San Diego, CA), 10% FBS, 100 U/ml penicillin, and 100 µg/ml streptomycin and were cultured in the presence or absence of 10 ng/ml IL‐33 (BioLegend) for 4 days. The concentrations of IL‐5 and IL‐13 in cell‐free supernatants were measured using a MILLIPLEX MAP Human Cytokine/Chemokine Panel from EMD Millipore (Billerica, MA). The minimal detection limits for IL‐5 and IL‐13 are 3.2 pg/ml.

### Statistics

All data were reported as the median (25–75% interquartiles) or as the mean ± SEM. Differences between groups were analyzed using the 1‐way ANOVA Kruskal–Wallis Dunn's multiple comparison test, the Mann‐Whitney test, or the Paired *t‐*test. A *p*‐value of less than 0.05 was considered significant.

## Results

### ILC2 are elevated in CRSwNP

**Figure 1:**
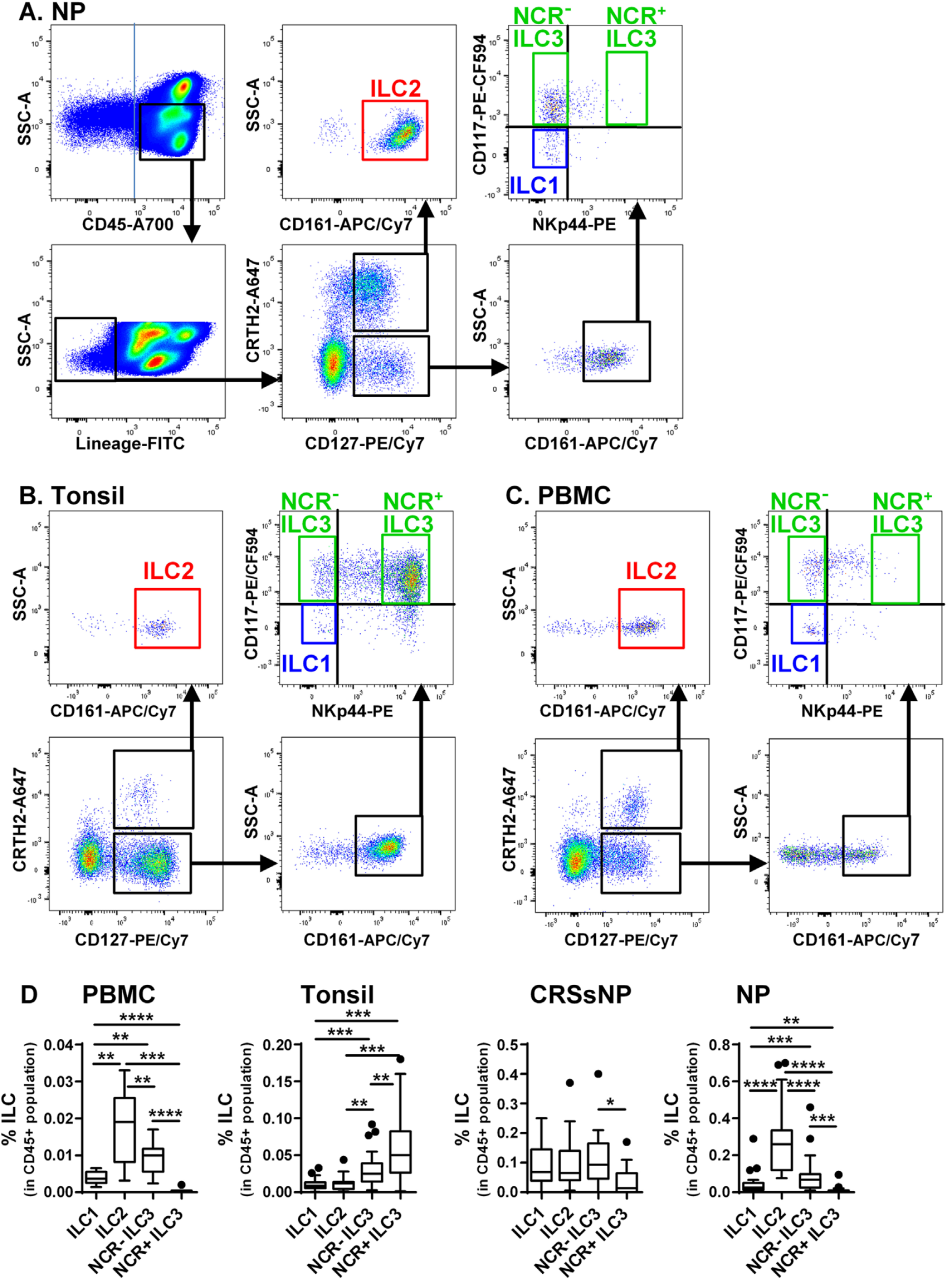
Characterization of ILC subsets. Representative flow cytometric plots for ILC subsets in NP are shown within the singlets and Aqua‐population. We selected the CD45^+^ population, excluded granulocytes (SSChigh), selected the Lin (CD3, CD11c, CD14, CD16, CD19, CD20, CD56, FcϵR1a) negative population, and then identified ILC subsets by the following markers; ILC1 (CD127^+^, CRTH2^−^, CD161^+^, CD117^−^, NKp44^−^), ILC2 (CD127^+^, CRTH2^+^, CD161^+^), NCR^−^ ILC3 (CD127^+^, CRTH2^‐^, CD161^+^, CD117^+^, NKp44^−^) and NCR^+^ ILC3 (CD127^+^, CRTH2^−^, CD161^+^, CD117^+^, NKp44^+^) (A). Representative flow cytometric plots for ILC subsets in tonsil and PBMC are shown within the singlets, Aqua^−^, CD45^+^, Lin^−^, and granulocyte excluded population (B and C). The frequency of ILC subsets in the total CD45^+^ population in PBMC (*n* = 12), tonsil (*n* = 21), CRSsNP (*n* = 13) and NP (*n* = 25) were calculated (D). *p < 0.05, **p < 0.01, ***p < 0.001, ****p < 0.0001, by one‐way ANOVA.

To characterize ILC subsets, we collected ethmoid tissue from CRSsNP, NPs from CRSwNP, tonsil tissue from patients undergoing tonsillectomy and PBMC from healthy subjects, and determined the presence and frequency of ILC in the CD45^+^ cell population by flow cytometry based on a gating strategy developed by Hazenberg and Spits 4 with some modifications. We first selected the singlets and the Aqua^−^ live cell population and then selected the CD45^+^ population, excluded granulocytes (side scatter (SSC) high), and selected the Lin (CD3, CD11c, CD14, CD16, CD19, CD20, CD56, FcϵR1a) negative population (Fig. 1). In order to determine a cut off line for the Lin^−^ population, we used anti‐CD4 or anti‐CD5 instead of anti‐ICOS and anti‐KLRG1 in a second panel (not shown). We then identified ILC subsets using the following markers; ILC1 (CD127^+^, CRTH2^−^, CD161^+^, CD117^−^, NKp44^−^), ILC2 (CD127^+^, CRTH2^+^, CD161^+^), NCR^−^ ILC3 (CD127^+^, CRTH2^−^, CD161^+^, CD117^+^, NKp44^−^) and NCR^+^ ILC3 (CD127^+^, CRTH2^−^, CD161^+^, CD117^+^, NKp44^+^) (Fig. 1). We found that all ILC subsets were present in PBMC, tonsil and CRS but the frequency was tissue dependent (Fig. 1). ILC2 were the major type in PBMC and NP while NCR^+^ ILC3 were dominant in tonsil (Fig. 1). In contrast, the frequency of ILC subsets was similar, except for NCR^+^ ILC3, in CRSsNP (Fig. 1).

**Figure 2:**
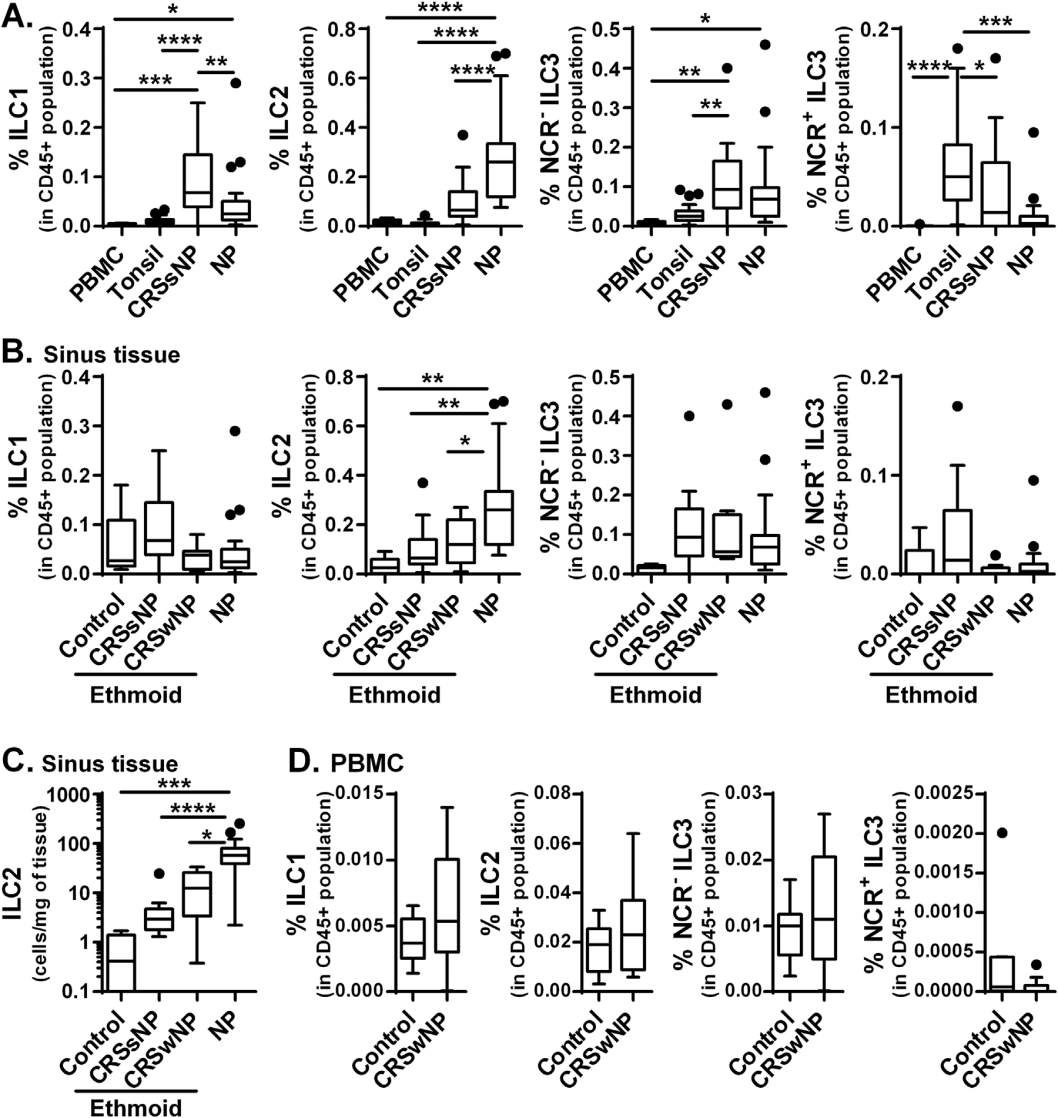
ILC2 are elevated in local CRSwNP tissue. We re‐plotted the frequency of ILC within each ILC subset (A). The frequency of ILC subsets in the total CD45^+^ population in PBMC (*n* = 12), tonsil (*n* = 21), CRSsNP (*n* = 13), and NP (*n* = 25) were calculated (A). We also collected ethmoid sinus tissues from control subjects (*n* = 5), and patients with CRSwNP (*n* = 9) and the frequency of ILC subsets in the total CD45^+^ population were calculated (B). Numbers of ILC2 in sinus tissues were normalized by mg of tissue (C). The frequency of ILC subsets in the total CD45^+^ population in PBMC from healthy control subjects (*n* = 12, data is from Figs. 1D and 2A) and CRSwNP (*n* = 13) were calculated (D). *p < 0.05, **p < 0.01, ***p < 0.001, ****p < 0.0001, by one‐way ANOVA.

We next compared the frequency of ILC between groups. We found that the frequency of ILC1 was significantly elevated in CRSsNP compared to PBMC, tonsil, and NP (Fig. 2A). The frequency of NCR^−^ ILC3 was also significantly higher in CRSsNP than in PBMC and tonsil (Fig. 2A). In contrast, NCR^+^ ILC3 were significantly elevated in tonsils (Fig. 2A). We then focused on ILC2 which are predominant in NP. We found that the frequency of ILC2 was significantly elevated in NP compared to PBMC, tonsil, and CRSsNP (Fig. 2A).

Next we compared the frequency of ILC subsets between NP tissue and ethmoid sinus tissues from control, CRSsNP and CRSwNP in a small cohort. We found that there were no differences in the frequency of ILC1 or ILC3 between groups (Fig. 2B). In contrast, the frequency of ILC2 was elevated in NP compared to ethmoid tissues from control, CRSsNP or CRSwNP (Fig. 2B). Since inflammatory cells were more accumulated in CRSwNP, we also normalized the data by weight of the tissue. We found that ILC2 numbers were also significantly elevated in NP (Fig. 2C). Although CRSwNP ethmoid tissues showed 23‐fold higher levels of ILC2 compared to control ethmoid tissue, this difference did not reach significance (Fig. 2C). This was probably due to the sample size of the current study and it will require further study with a larger cohort to test the hypothesis that ILC2 are also elevated in non polyp areas of CRSwNP. Taken together, these results confirmed published studies 5, 6, 7, 8, 9 that ILC2 are highly accumulated in CRSwNP.

To test whether accumulation of ILC2 in CRSwNP patients can be found systemically, we also isolated PBMC from CRSwNP patients and compared them to control subjects. We found that there was no significant difference in the frequency of ILC subsets when PBMC from healthy subjects and patients with CRSwNP were compared (Fig. 2D). This suggests that the frequency of ILC subsets in CRS is regulated locally. However, a recent study showed that peripheral blood ILC2 were increased during pollen season in patients with allergic rhinitis 14. The determination of seasonal changes of blood ILC2 may require in future a study with a larger cohort. Since 52% of CRSwNP patients took oral and/or nasal steroid, 68% of CRSwNP were comorbid with asthma and 24% have aspirin exacerbated respiratory disease (AERD), we examined whether this affected the frequency of ILC2 in CRSwNP. However, we found no significant difference in ILC2 levels based on the status of steroid treatment, status of asthmatics or presence of AERD (Fig. S1).

### Differential expression of CD127 and ICOS on ILC2 in CRSwNP

**Figure 3:**
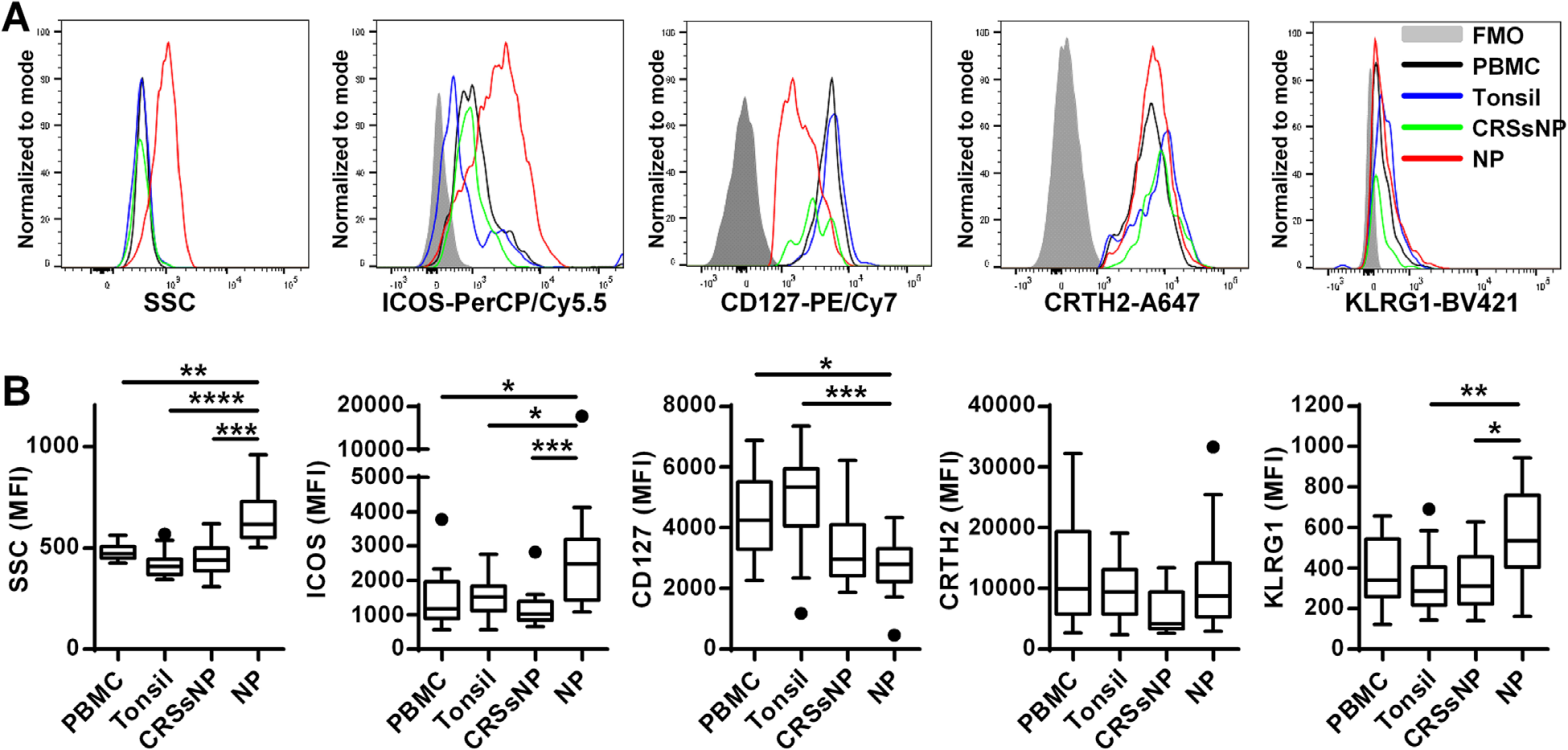
Altered expression of CD127 and ICOS on ILC2 in NPs. Representative histograms of flow cytometric plots for SSC, ICOS, CD127, CRTH2, and KLRG1 in ILC2 from a PBMC, a tonsil, a CRSsNP and a CRSwNP are shown (A). Levels on ILC2 from PBMC (*n* = 12), tonsils (*n* = 21), CRSsNP (*n* = 13), and NP (*n* = 25) are shown by mean fluorescence intensity (MFI) (B). *p < 0.05, **p < 0.01, ***p < 0.001, ****p < 0.0001, by one‐way ANOVA.

Since CRSwNP is characterized by type 2 inflammation and ILC2 can produce large amounts of type 2 cytokines, we hypothesized that ILC2 are not only elevated but also activated in CRSwNP. Recently, Salimi et al. found the up‐regulation of killer cell lectin like receptor G1 (KLRG1) on ILC2 in atopic dermatitis, and Maazi et al. reported that inducible T‐cell co‐stimulator (ICOS) on ILC2 controlled type 2 inflammation 15, 16. Therefore, we first examined the expression of KLRG1, ICOS and ILC2 markers in ILC2 from PBMC, tonsil, and CRS. When we compared NP ILC2 to PBMC, tonsil and CRSsNP ILC2, we found that the levels of side scatter (SSC) and ICOS were increased, CD127 (except in comparison with CRSsNP) was down‐regulated but CRTH2 did not show a difference (Fig. 3). In addition, KLRG1 was weakly up‐regulated in NP ILC2 compared to tonsil and CRSsNP ILC2 (Fig. 3B). In contrast, there was no difference in the level of marker expression on ILC2 between PBMC, tonsils and CRSsNP (Fig. 3). This result suggests that ILC2 in CRSwNP might be subject to several micro environmental factors within the inflamed area potentially changing expression levels of cell surface molecules.

### TSLP and IL‐33 down‐regulate cell surface expression of CD127 on ILC2

**Figure 4:**
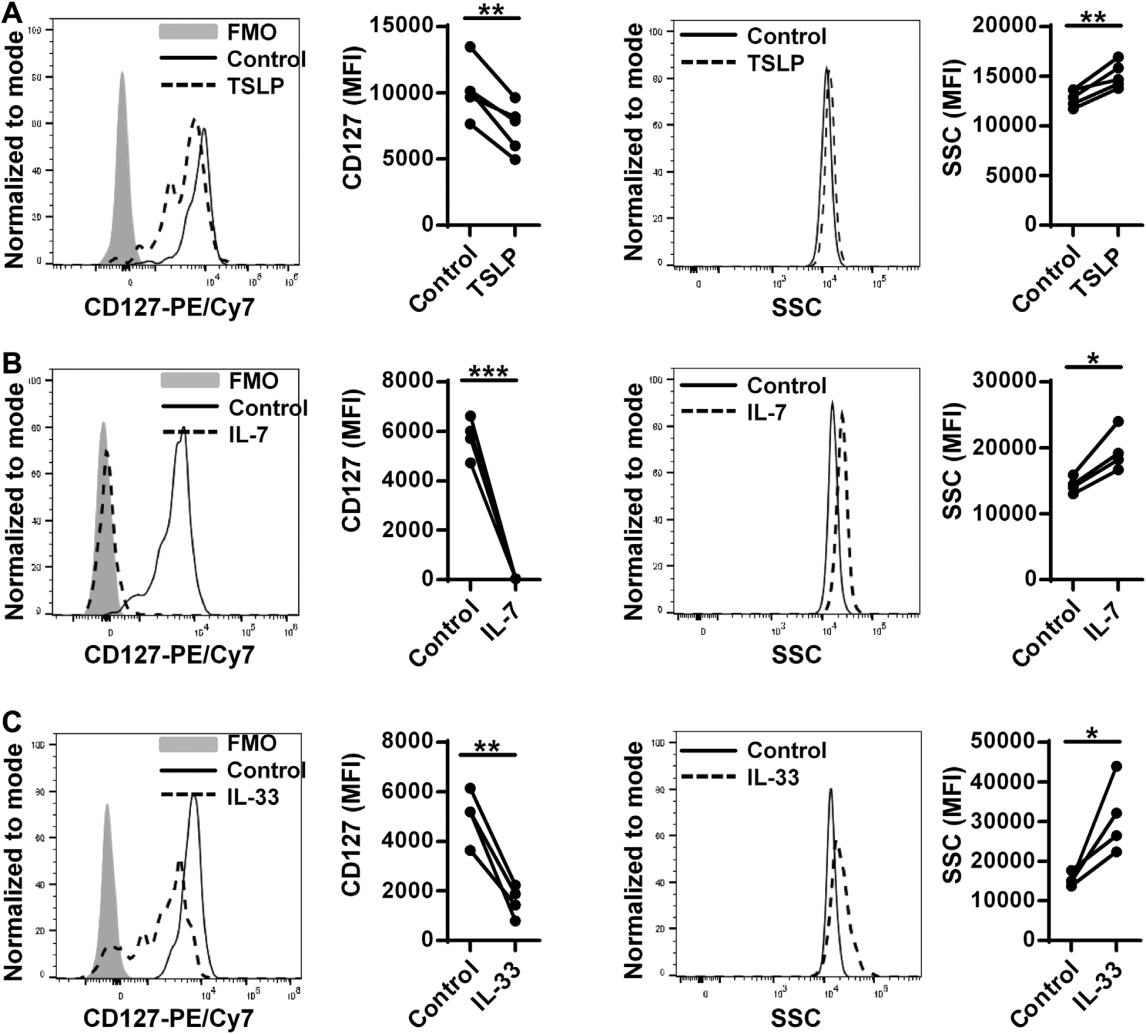
The level of CD127 and SSC is altered in activated ILC2. PBMC were stimulated with medium control, 10 ng/ml TSLP (A. *n* = 5) or 10 ng/ml IL‐7 (B. *n* = 4) for two days. Sorted blood ILC2 were cultured in the presence or absence of 10 ng/ml IL‐33 for four days (C. *n* = 4). Representative histograms of flow cytometric plots for CD127 and SSC in ILC2 from FMO control (filled), medium control culture (solid line) and cytokine stimulation (dashed line) were shown. Levels of CD127 and SSC on ILC2 are shown by mean fluorescence intensity (MFI). *p < 0.05, **p < 0.01, ***p < 0.001 by paired *t*‐test.

CD127 (also known as IL‐7Ra) is the α subunit of the receptor for both IL‐7 and thymic stromal lymphopoietin (TSLP) and the TSLP receptor complex is expressed on ILC2 4. We recently found that TSLP is upregulated in CRSwNP 17. We investigated the potential role of TSLP and IL‐7 on the cell surface expression of CD127 using blood ILC2. We found that TSLP and IL‐7 were able to down‐regulate cell surface expression of CD127 in blood ILC2 (Fig. 4A and B). Interestingly, they also increased the level of SSC on blood ILC2 (Fig. 4A and B). In addition to TSLP, IL‐33 has been reported present in CRSwNP tissue 6. IL‐33 is well known to act as a potent stimulus on ILC2 inducing the production of type 2 cytokines including IL‐5 and IL‐13 accompanied by morphological changes 5, 18. We next investigated the effect of IL‐33 on the levels of CD127 and SSC. We found that IL‐33 down‐regulated CD127 and enhanced levels of SSC in blood ILC2 (Fig. 4C). These results indicate that the levels of CD127 and SSC are changed in activated ILC2, and ILC2 in NPs may be activated in part due to the presence of TSLP and IL‐33 in NPs.

### ILC2 are activated in CRSwNP

**Figure 5:**
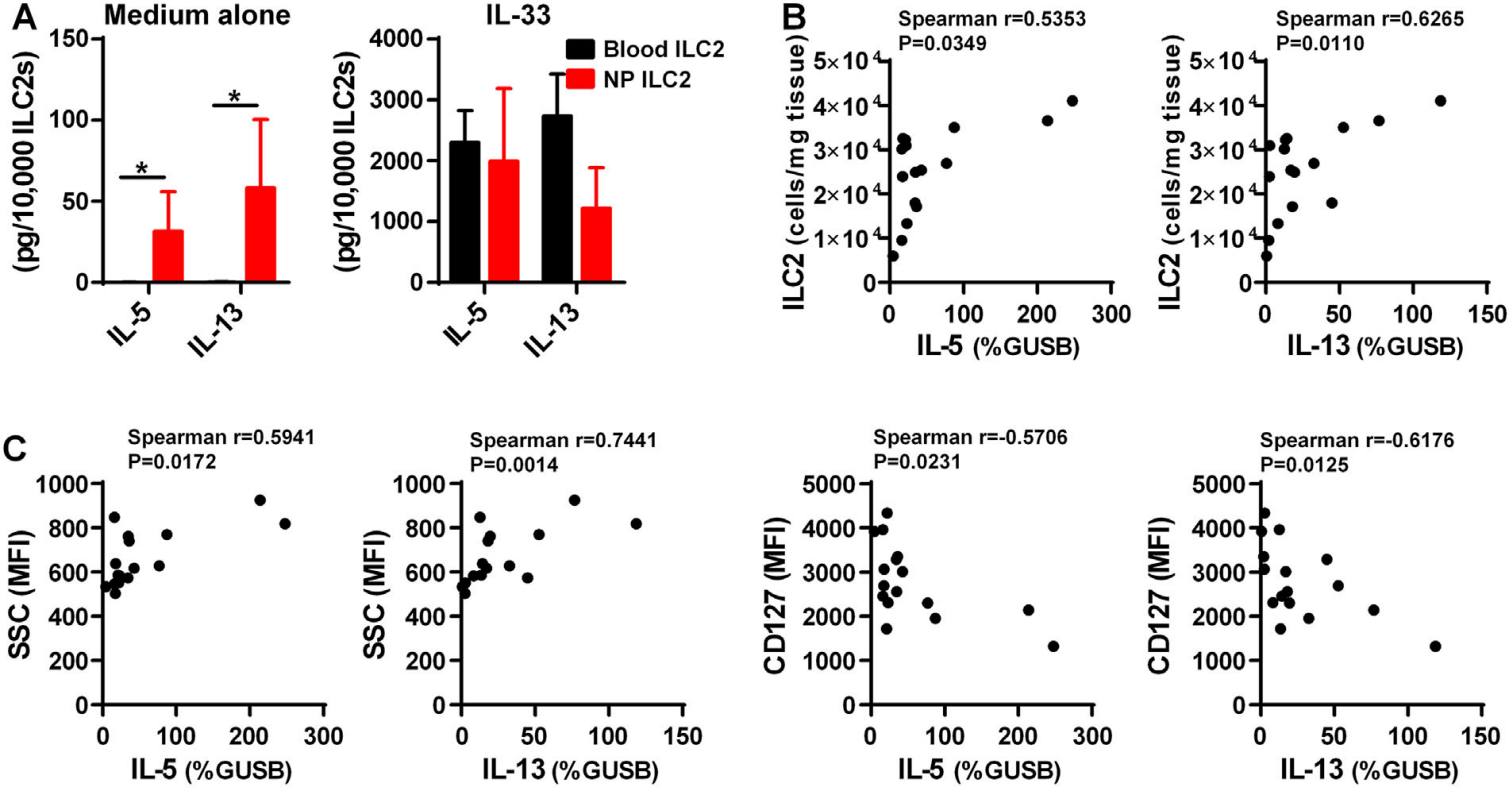
ILC2 are activated and release IL‐5 and IL‐13 in NPs. Sorted blood ILC2 (black, *n* = 4) and NP ILC2 (red, *n* = 4) were cultured in the presence or absence of 10 ng/ml IL‐33 for four days (A). The concentrations of IL‐5 and IL‐13 were measured by using Luminex. (B) shows correlations between gene expression and concentration of ILC2 in NP tissues, and (C) shows correlations between gene expression in NP tissue and levels of SSC and CD127 on NP ILC2. Concentration of ILC2 in NP tissues and levels of SSC and CD127 on NP ILC2 were assessed by flow cytometry and mRNAs for IL‐5 and IL‐13 in whole NP tissue were assessed by real‐time RT‐PCR (*n* = 16). Gene expression levels were shown as % expression of the housekeeping gene β‐glucuronidase (GUSB). The correlations were assessed using Spearman rank correlation.

To further investigate whether ILC2 are truly activated in NP in vivo, we isolated ILC2 from the blood of healthy subjects and from NP tissue of CRSwNP patients and compared the production of type 2 cytokines. Interestingly, NP ILC2 but not blood ILC2 spontaneously released IL‐5 and IL‐13 without additional stimulation, although a common ILC2 activator, IL‐33, was able to induce production of type 2 cytokines from both blood and NP ILC2 (Fig. 5A). This result indicates that ILC2 are already activated in CRSwNP and indeed produce type 2 cytokines in vivo.

We next investigated whether the accumulation of ILC2 affects type 2 inflammation in NPs. We isolated RNA from the same NP tissues and examined the levels of type 2 cytokines. We found that concentration of ILC2 correlated with tissue IL‐5 and IL‐13 in NPs (Fig. 5B). We also investigated whether the observed changes of SSC, CD127, and ICOS in ILC2 affect type 2 inflammation in NPs. We found that levels of SSC and CD127 but not ICOS, CRTH2 nor KLRG1 on ILC2 correlated with tissue IL‐5 and IL‐13 in CRSwNP (Fig. 5C and S2). We also found that levels of SSC and CD127 on sorted ILC2 were significantly correlated with spontaneous production of IL‐5 and IL‐13 (Fig. S3), although this data was from only a small cohort. These results indicate that ILC2 are activated in NPs and level of CD127 on ILC2 may be used as a marker for activation status of ILC2 in CRSwNP, although the use of SSC as a phenotypical cut off for the ILC2 population might be difficult.

## Discussion

Although animal models have elegantly demonstrated the importance of ILC in immune responses and inflammatory diseases, the investigation of roles for ILC in human systems has been hampered by technical limitations. Animal models have clearly demonstrated roles for ILC2 in type 2 inflammation and allergic inflammatory diseases 19, 20, 21, 22. In contrast, there are limited reports elucidating the role of ILC2 in human diseases and most studies have shown only the presence and elevation of ILC2 in type 2 inflammatory diseases including CRSwNP, asthma, and atopic dermatitis 5, 6, 7, 8, 9, 15, 18, 23. Recent studies have shown that environmental factors and cytokines can induce plasticity of ILC2 24, 25, 26. These results indicate the importance of investigation into the presence of ILC1, ILC3, factors that control plasticity of ILC2, as well as phenotypical differences of ILC2 in type 2 inflammatory diseases. However, to the best of our knowledge, the presence of ILC1, ILC3, and the phenotype of ILC2 in CRSwNP have not been carefully studied although the presence of ILC1 and ILC3 in CRS was implicated by a meeting abstract 27. Here we found that all ILC subsets were present in CRSwNP but the frequency of ILC1, NCR^−^ ILC3 and NCR^+^ ILC3 was significantly less than ILC2 by 6.2, 3.1, and 29.8‐fold, respectively, in NPs (Fig. 1D). In addition, ILC2 were significantly elevated in NPs compared to PBMC, tonsil and sinus tissues from control patients and CRSsNP patients (Fig. 2). These results suggest that recruitment, maturation, and differentiation factors of ILC2 might be elevated but factors that control ILC1 or ILC3 transition from ILC2 might be reduced in NPs. Indeed although IL‐12 is able to convert human ILC2 into an ILC1‐like phenotype 24, 25, 26, IL‐12 and IFN‐γ are not elevated in CRSwNP 3, 17, 28, 29. Currently, the factors that control transition of ILC2 into ILC3 and recruitment factors of ILC2 have not been identified and future study will be required to identify these factors and measure their presence in CRSwNP.

Although ILC2 are known to be elevated in NPs, it was not clear whether the accumulated ILC2 were activated or not. We initially selected KLRG1 and ICOS as potential markers of activation of ILC2. We found that ICOS was significantly elevated in NP ILC2 compared to ILC2 from PBMC, tonsil and CRSsNP (Fig. 3). We then investigated the correlation of levels of ICOS on CRSwNP ILC2 with levels of IL‐5 and IL‐13 in tissue. However, there was no correlation between ICOS and type 2 cytokines (Fig. S2). Although ICOS on ILC2 is known to control airway hyperreactivity and lung inflammation 16, its role in CRSwNP is still unclear. In addition to ICOS, we also found that CD127 was reduced and SSC was increased in CRSwNP ILC2 (Fig. 3). Interestingly, levels of CD127 on CRSwNP ILC2 were negatively and levels of SSC in CRSwNP ILC2 were positively correlated with tissue type 2 inflammation (Fig. 5C). Importantly we also found that levels of CD127 and SSC significantly correlated with the spontaneous production of IL‐5 and IL‐13 in sorted ILC2 (Fig. S3). These results suggest that ILC2 in NPs may be phenotypically altered compared to ILC2 in blood, tonsil and CRSsNP and also that ILC2 were already activated and releasing type 2 cytokines including IL‐5 and IL‐13 in NPs.

Since the changes of CD127 and SSC on ILC2 from tissue were novel and they correlated with type 2 inflammation, we further investigated whether they were triggered by activation. We found that IL‐7, IL‐33, and TSLP were able to reduce cell surface expression of CD127 and to increase SSC in blood ILC2 (Fig. 4). We first focused on IL‐7 and found that down‐regulation of CD127 by IL‐7 was due to internalization of the receptor (Fig. S4A). However, our data indicates that down‐regulation of CD127 in NP ILC2 was not only due to internalization (Fig. S4B). In addition, we could not find the up‐regulation of IL‐7 in NPs based on our published microarray study 30. This suggests that IL‐7 may not play a major role in the down‐regulation of CD127 on ILC2 in NP tissue. In contrast, the presence of TSLP and IL‐33 in NPs have been implicated by several investigators 6, 17, 31. IL‐33 and TSLP are known to activate different signaling pathways 32, 33. This suggests that changes of CD127 and SSC may be a common event during the activation of ILC2 and that TSLP and IL‐33 may not be the only factors to control the function of ILC2 in CRSwNP. Further study will be required to identify the mechanisms of activation of ILC2 in CRSwNP. Most importantly, CD127 is a marker commonly used to identify ILC2 but is down‐regulated in CRSwNP. This strongly suggests that the current gating strategy may exclude some of the activated ILC2 and that the frequency and presence of ILC2 in CRSwNP or other type 2 inflammatory diseases may actually be much higher than currently reported. Further studies will be required to identify common markers of ILC2 that are not affected by activation.

To the best of our knowledge, this is the first comprehensive report showing the presence of all known ILC subsets in CRS. Our data indicates that ILC2 are predominant in CRSwNP and are elevated in CRSwNP compared to blood, tonsil, and CRSsNP. We also present the first evidence that ILC2 are activated in CRSwNP in vivo based on the expression of cell surface molecules and spontaneous production of type 2 cytokines from freshly isolated ILC2. Our findings indicate that ILC2 may play a significant role in type 2 inflammation in CRSwNP and that pathways of recruitment and activation of ILC2 in CRSwNP may indicate new therapeutic strategies for this disease. These pathways in CRSwNP are worthy of further investigation.

## Authors’ Contributions

AK designed the study. JAP, AIK, and AK performed the experiments. AK analyzed the data. BKT, PS, HB, GL, KEH, WWS, ATP, LCG, RPS, KCW, SSS, DBC, JRR, JGK, OA, and RCK helped in sample collection and evaluation. AK and JAP wrote the manuscript. All authors have read and approved the final form of the manuscript.

## Conflicts of Interest

The authors declare no conflict of interest as to the interpretation and presentation of this manuscript.

## Supporting information

Additional supporting information may be found in the online version of this article at the publisher's web‐site.

Supporting Data S1.

**Figure S1**. Steroid treatment, asthmatic status or presence of aspirin sensitivity did not affect the levels of ILC2 in NPs. The frequency of ILC subsets in the total CD45+ population in NPs (*n* = 25) was determined by flow cytometry. We compared the presence of ILC2 in NPs by history of glucocorticoid (GC) treatment (none (*n* = 12), nasal GC (*n* = 4), oral GC (*n* = 4), nasal and oral GC (*n* = 5)), asthmatic status (non asthmatic (*n* = 8), asthmatic (*n* = 17)), or presence of aspirin exacerbated respiratory disease (AERD) (non AERD (*n* = 19), AERD (*n* = 6)). There were no differences by one‐way ANOVA.

**Figure S2**. Correlation between phenotype of ILC2 and level of IL‐5 in NPs. Levels of ICOS, CRTH2 and KLRG1 on NP ILC2 were determined by flow cytometry and mRNA for IL‐5 in NP tissue was assessed by real‐time RT‐PCR. Gene expression levels were shown as % expression of housekeeping gene GUSB. The correlations were assessed by using Spearman rank correlation (*n* = 16).

**Figure S3**. Levels of SSC and CD127 correlate with spontaneous production of IL‐5 and IL‐13 in ILC2. Sorted blood ILC2 (black, *n* = 4) and NP ILC2 (red, *n* = 4) were cultured in the absence of IL‐33 for 4 days. The concentrations of IL‐5 and IL‐13 were measured by using Luminex and the levels of SSC and CD127 on ILC2 by flow cytometry.

**Figure S4**. Reduction of cell surface CD127 in NP ILC2 may not be due to internalization. PBMCs were stimulated with medium control or 10 ng/ml IL‐7 for 2 days and ILC2 were detected by flow cytometry (A). Cells were isolated from NP tissue and NP ILC2 were detected by flow cytometry (B). ILC2s were stained by anti‐CD127 antibody (clone ebioRDR5) before and after permeabilization.
