# Supplementary material for: Group 2 innate lymphoid cells are elevated and activated in chronic rhinosinusitis with nasal polyps
Source: Immun Inflamm Dis. 2017 Apr 19;5(3):233–43. doi: 10.1002/iid3.161 (PMC5569375; doi:10.1002/iid3.161)
Supplement: Supplementary file 1 — Supporting Data S1. [file IID3-5-233-s001.docx]

Supporting information

**Group 2 innate lymphoid cells are elevated and activated in chronic rhinosinusitis with nasal polyps**

**Julie A. Poposki, MS^a^; Aiko I. Klingler, PhD^a^; Bruce K. Tan, MD, MS^b^; Pejman Soroosh, PhD^c^; Homayon Banie, PhD^c^; Gavin Lewis, PhD^c^; Kathryn E. Hulse, PhD^a^; Whitney W. Stevens, MD, PhD^a^; Anju T. Peters, MD^a^; Leslie C. Grammer, MD^a^; Robert P. Schleimer, PhD^a,b^; Kevin C. Welch, MD^b^; Stephanie S. Smith, MD^b^; David B. Conley, MD^b^; Joseph R. Raviv, MD^d^; James G. Karras, PhD^c^; Omid Akbari, PhD^e^; Robert C. Kern, MD^a,b^; Atsushi Kato, PhD^a, b, *^.**

^a^ Division of Allergy-Immunology, Department of Medicine, Northwestern University Feinberg School of Medicine, Chicago, IL 60611, USA.

^b^ Department of Otolaryngology, Northwestern University Feinberg School of Medicine, Chicago, IL 60611, USA.

^c^ Janssen Research and Development, San Diego, CA 92121, USA.

^d^ Division of Otolaryngology-Head and Neck Surgery, NorthShore University HealthSystem, The University of Chicago, Pritzker School of Medicine, Evanston, IL 60201, USA.

^e^ Department of Molecular Microbiology and Immunology, Keck School of Medicine, University of Southern California, Los Angeles, Los Angeles, CA 90033, USA

**Materials and methods**

***Cell isolation for flow cytometric analysis***

Tissue samples obtained during surgery were weighed, washed by dPBS, fragmented and then incubated with 30 µg/ml DNase I and 1 mg/ml type I collagenase containing media at 4^o^C overnight. Following this, tissues were minced using a gentleMACS dissociator (Miltenyi Biotec, Auburn, CA) and the cells were filtered through 70 µm nylon mesh (BD Biosciences, San Jose, CA). Cells were then treated with red blood cell lysis solution (Miltenyi Biotec) and washed with dPBS before counting and staining for flow cytometric analysis.

***ILC2 sorting***

After the isolation of cells from NP tissues, cells were first treated with Aqua dead cell staining reagent. Cells were blocked by Fc Block reagent and then incubated with FITC anti-human Lineage Cocktail (CD3, CD14, CD16, CD19, CD20, CD56), FITC anti-FcεRIa, FITC anti-CD11c, Alexa Fluor 700 anti-CD45, Alexa Fluor 647 anti-CRTH2, PE/Cy7 anti-CD127 and APC/Cy7 anti-CD161. We sorted ILC2 as Aqua-, CD45^+^, Lin (FITC)^-^, CD127^+^, CRTH2^+^, CD161^+^ cells with a BD FACSAria SORP-5-laser cell sorter (BD Biosciences) at the Robert H Lurie Comprehensive Cancer Center at Northwestern University. For blood ILC2, human PBMC were isolated from a human peripheral blood leuko pak by centrifugation on a Ficoll-Paque PREMIUM density gradient (GE Healthcare, Piscataway, NJ). After lysis of red blood cells by red blood cell lysis solution (Miltenyi Biotec), PBMC were incubated with an Fc Block reagent and FITC anti-human Lineage Cocktail for 15 minutes at 4^o^C in the dark. FITC^+^ (Lin^+^) cells were depleted by human FITC positive selection kit (STEMCELL Technologies) using Big EasySep Magnets (STEMCELL Technologies). After depletion of Lin^+^ cells, cells were counted and further stained with FITC anti-FcεRIa, FITC anti-CD11c, Alexa Fluor 700 anti-CD45, Alexa Fluor 647 anti-CRTH2, PE/Cy7 anti-CD127 and APC/Cy7 anti-CD161. We then sorted ILC2 as CD45^+^, Lin (FITC)^-^, CD127^+^, CRTH2^+^, CD161^+^ cells with a BD FACSAria SORP-5-laser cell sorter. The purity of ILC2 was always greater than 95% (not shown).

***Real-time RT-PCR***

A portion of NP tissue for isolation of RNA was transferred in RNAlater (Ambion, Austin, TX) and stored at -20°C. Total RNA from sinus tissue was extracted using QIAzol (Qiagen, Valencia, CA) and was cleaned and treated with DNase I using NucleoSpin RNA kits (Clontech Laboratories, Mountain View, CA) according to the manufacturer's instructions. The quality of total RNA from sinus tissue was assessed with a 2100 Bioanalyzer (Agilent Technologies, Santa Clara, CA) using a RNA 6000 Nano LabChip (Agilent Technologies). We extracted RNA from 18 NP tissues and the 16 RNA samples in which RIN was greater than 7.0 were used for cDNA synthesis. Single-strand cDNA was synthesized with SuperScript II reverse transcriptase (Invitrogen, Carlsbad, CA) and random primers. Real-time RT-PCR was performed using the TaqMan method on a StepOnePlus Real-Time PCR System (Applied Biosystems, Foster City, CA) in 20 µl reactions (10 µl 2x TaqMan Fast Advanced Master Mix (Applied Biosystems), 1 µl 20x primer and probe mixture for target gene, 1 µl 20x primer and VIC/MGB probe mixture for β-glucuronidase (GUSB) plus cDNA equivalent to 10 ng of total RNA). Primer and probe sets for IL-5 (sense, 5'- AGCTGCCTACGTGTATGCCA-3'; antisense, 5'-GTGCCAAGGTCTCTTTCACCA-3'; FAM/BHQ1 probe, 5'-CCCCACAGAAATTCCCACAAGTGCA-3'), IL-13 (sense, 5'-AAGGTCTCAGCTGGGCAGTTTA-3'; antisense, 5'-AAACTGGGCCACCTCGATT-3'; FAM/BHQ1 probe, 5'-CCAGCTTGCATGTCCGAGACACCA-3') and GUSB (Human β-glucuronidase endogenous control, PN; 4326320E) were purchased from Integrated DNA Technologies (Coralville, IA) or Applied Biosystems. The mRNA expression levels were normalized to the expression of a housekeeping gene, GUSB.

**Figures Legend**

**Figure S1.** **Steroid treatment, asthmatic status or presence of aspirin sensitivity did not affect the levels of ILC2 in NPs.**

The frequency of ILC subsets in the total CD45+ population in NPs (n=25) was determined by flow cytometry. We compared the presence of ILC2 in NPs by history of glucocorticoid (GC) treatment (none (n=12), nasal GC (n=4), oral GC (n=4), nasal and oral GC (n=5)), asthmatic status (non asthmatic (n=8), asthmatic (n=17)), or presence of aspirin exacerbated respiratory disease (AERD) (non AERD (n=19), AERD (n=6)). There were no differences by one-way ANOVA.

**Figure S2. Correlation between phenotype of ILC2 and level of IL-5 in NPs.**

Levels of ICOS, CRTH2 and KLRG1 on NP ILC2 were determined by flow cytometry and mRNA for IL-5 in NP tissue was assessed by real-time RT-PCR. Gene expression levels were shown as % expression of housekeeping gene GUSB. The correlations were assessed by using Spearman rank correlation (n=16).

**Figure S3. Levels of SSC and CD127 correlate with spontaneous production of IL-5 and IL-13 in ILC2.**

Sorted blood ILC2 (black, n=4) and NP ILC2 (red, n=4) were cultured in the absence of IL-33 for 4 days. The concentrations of IL-5 and IL-13 were measured by using Luminex and the levels of SSC and CD127 on ILC2 by flow cytometry.

**Figure S4. Reduction of cell surface CD127 in NP ILC2 may not be due to internalization.**

PBMCs were stimulated with medium control or 10 ng/ml IL-7 for 2 days and ILC2 were detected by flow cytometry (A). Cells were isolated from NP tissue and NP ILC2 were detected by flow cytometry (B). ILC2s were stained by anti-CD127 antibody (clone ebioRDR5) before and after permeabilization.
