# Supplementary figures and images for: Group 2 innate lymphoid cells are elevated and activated in chronic rhinosinusitis with nasal polyps
Source: Immun Inflamm Dis. 2017 Apr 19;5(3):233–43. doi: 10.1002/iid3.161 (PMC5569375; doi:10.1002/iid3.161)

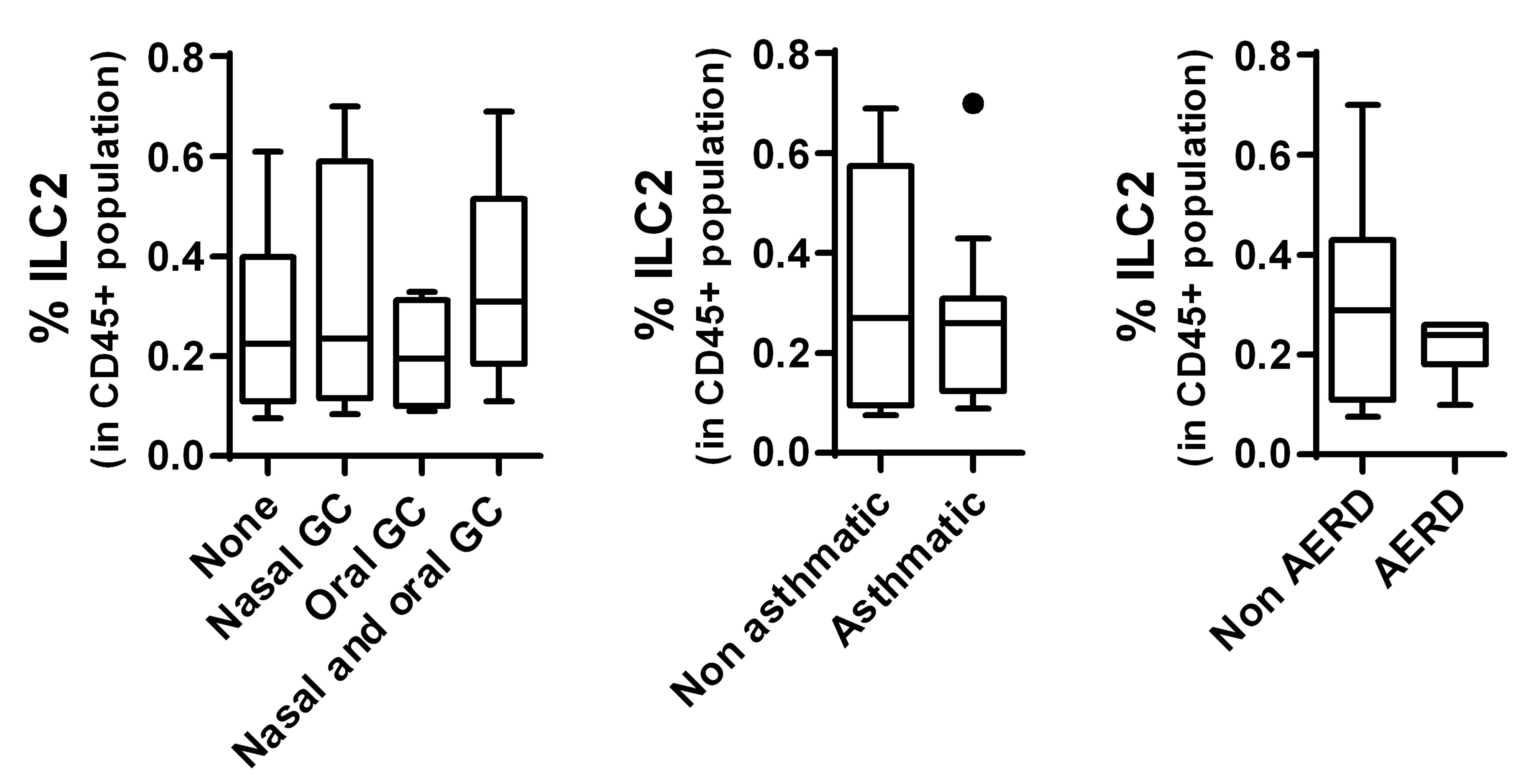

Supplement: Supplementary file 2 — Figure S1. Steroid treatment, asthmatic status or presence of aspirin sensitivity did not affect the levels of ILC2 in NPs. The frequency of ILC subsets in the total CD45+ population in NPs (n = 25) was determined by flow cytometry. We compared the presence of ILC2 in NPs by history of glucocorticoid (GC) treatment (none (n = 12), nasal GC (n = 4), oral GC (n = 4), nasal and oral GC (n = 5)), asthmatic status (non asthmatic (n = 8), asthmatic (n = 17)), or presence of aspirin exacerbated respiratory disease (AERD) (non AERD (n = 19), AERD (n = 6)). There were no differences by one‐way ANOVA. [file IID3-5-233-s002.tif]

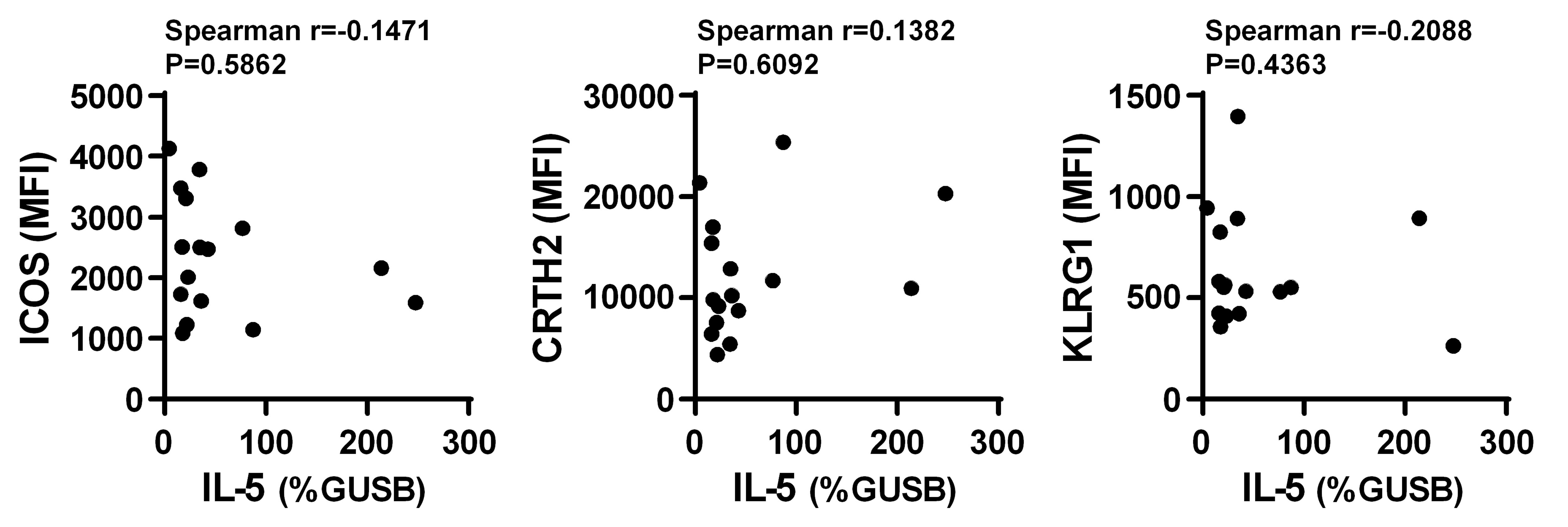

Supplement: Supplementary file 3 — Figure S2. Correlation between phenotype of ILC2 and level of IL‐5 in NPs. Levels of ICOS, CRTH2 and KLRG1 on NP ILC2 were determined by flow cytometry and mRNA for IL‐5 in NP tissue was assessed by real‐time RT‐PCR. Gene expression levels were shown as % expression of housekeeping gene GUSB. The correlations were assessed by using Spearman rank correlation (n = 16). [file IID3-5-233-s003.tif]

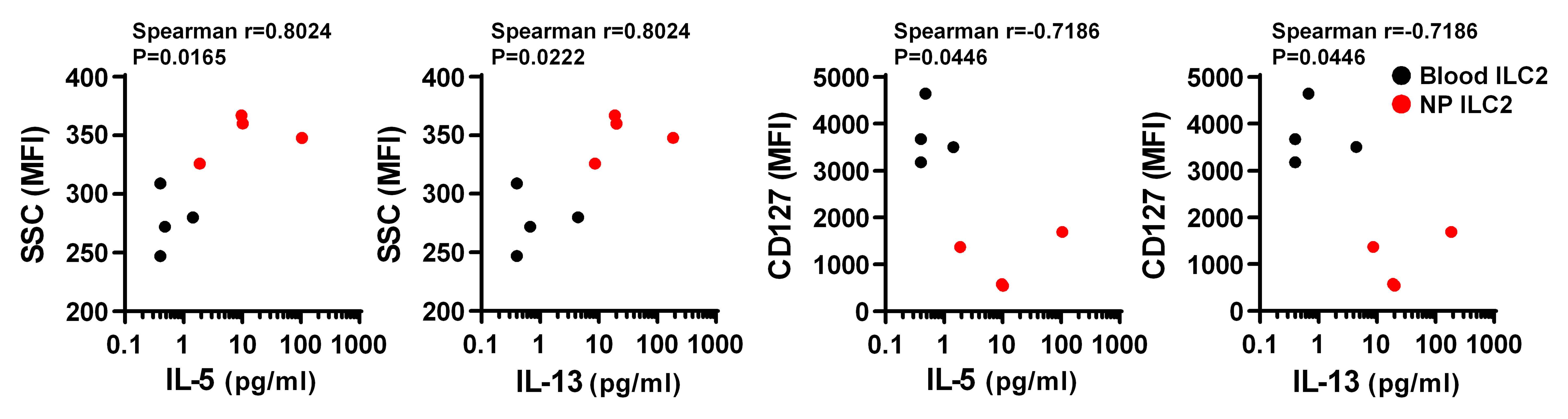

Supplement: Supplementary file 4 — Figure S3. Levels of SSC and CD127 correlate with spontaneous production of IL‐5 and IL‐13 in ILC2. Sorted blood ILC2 (black, n = 4) and NP ILC2 (red, n = 4) were cultured in the absence of IL‐33 for 4 days. The concentrations of IL‐5 and IL‐13 were measured by using Luminex and the levels of SSC and CD127 on ILC2 by flow cytometry. [file IID3-5-233-s004.tif]

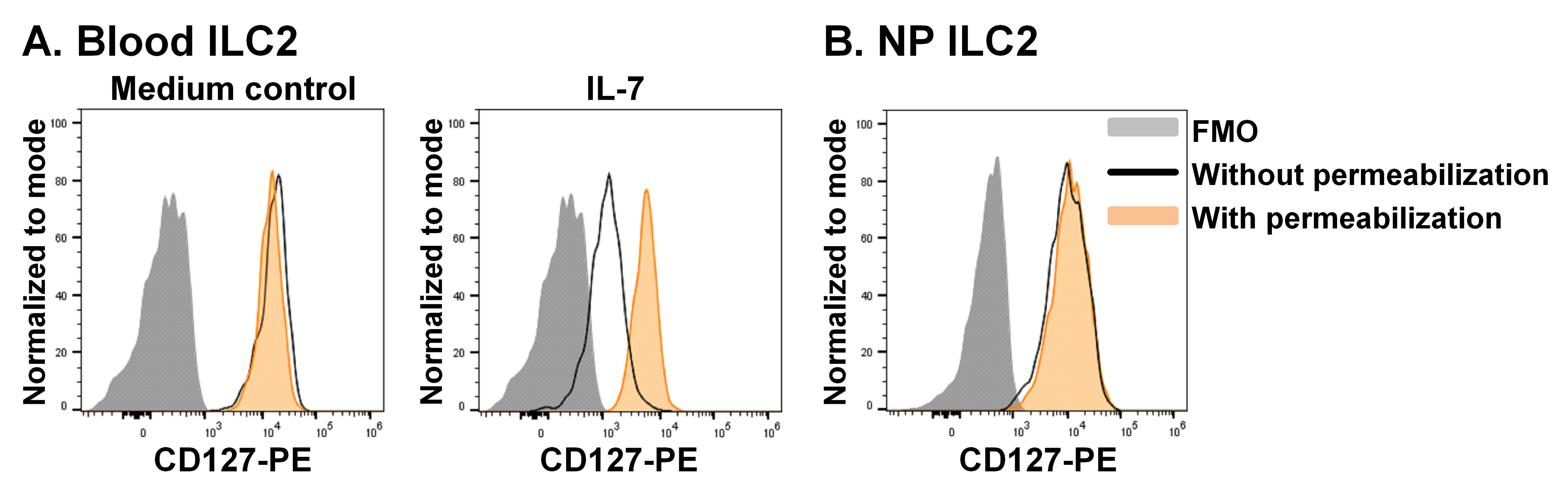

Supplement: Supplementary file 5 — Figure S4. Reduction of cell surface CD127 in NP ILC2 may not be due to internalization. PBMCs were stimulated with medium control or 10 ng/ml IL‐7 for 2 days and ILC2 were detected by flow cytometry (A). Cells were isolated from NP tissue and NP ILC2 were detected by flow cytometry (B). ILC2s were stained by anti‐CD127 antibody (clone ebioRDR5) before and after permeabilization. [file IID3-5-233-s005.tif]
